# Implicit learning of *what* comes *when* and *where* within a sequence: The time-course of acquiring serial position-item and item-item associations to represent serial order

**DOI:** 10.2478/v10053-008-0106-0

**Published:** 2012-05-21

**Authors:** Nicolas W. Schuck, Robert Gaschler, Peter A. Frensch

**Affiliations:** 1 Department of Psychology, Humboldt-Universität zu Berlin, Germany; 2 Max Planck Institute for Human Development, Center for Lifespan Psychology, Berlin, Germany

**Keywords:** implicit sequence learning, serial order, SRT, chaining, race model

## Abstract

Much research has been conducted aimed at the representations and mechanisms that enable learning of sequential structures. A central debate concerns the question whether item-item associations (i.e., in the sequence *A-B-C-D*, *B* comes after *A*) or associations of item and serial list position (i.e., *B* is the second item in the list) are used to represent serial order. Previously, we showed that in a variant of the implicit serial reaction time task, the sequence representation contains associations between serial position and item information (Schuck, Gaschler, Keisler, & Frensch, 2011). Here, we applied models and research methods from working memory research to implicit serial learning to replicate and extend our findings. The experiment involved three sessions of sequence learning. Results support the view that participants acquire knowledge about order structure (item-item associations) and about ordinal structure (serial position-item associations). Analyses suggest that only the simultaneous use of the two types of knowledge acquisition can explain learning-related performance increases. Additionally, our results indicate that serial list position information plays a role very early in learning and that inter-item associations increasingly control behavior in later stages.

## Introduction

The ability to flexibly store and retrieve sequential structures is fundamental to human cognition and ubiquitous in human behavior, such as in language or skill acquisition. The major theoretical challenge - the problem of serial order - in this field is twofold: first, to explain how a largely parallel system like the brain can store and produce sequentially ordered outputs (e.g., Houghton & Hartley, 1995). Second, the flexibility of serial memory/actions one can observe in humans seems to rule out traditional memory accounts that exclusively rely on associations between successive items (so called *chaining*; see Lashley, 1951). Consequently, the question of how the order and timing of events can be computed, stored, and retrieved has been investigated in a variety of different research contexts, such as working memory (e.g., Botvinick & Watanabe, 2007; Burgess & Hitch, 1999, 2006; Henson, 1998), motor learning (e.g., Salinas, 2009; Tanji, 2001), long-term memory (e.g., Howard & Kahana, 2002; Nairne, 1992), interval timing (e.g., Ivry & Spencer, 2004; Meck, Penney, & Pouthas, 2008), numerical cognition (e.g., Nieder, 2005; Verguts & Fias, 2006), and sequence learning in animals (e.g., Burns & Dunkman, 2000; Terrace, 2005). All this work is related to the issue of whether representations of the position of an item within a list (e.g., *B* is the second item in a list) are necessary to explain sequence representation, or if associations between successive items (e.g., *B* comes after *A*) are adequate as the sole mechanism. In a nutshell, the debate has been focused on the question what is the functional stimulus in serial learning and memory, the preceding action or the serial position/time of the action (Young, 1962; Young, Hakes, & Hicks, 1967)?

Consider the following example illustrating the difference between the two main classes of theories - those assuming the use of positional codes and those assuming inter-item associations: In a working memory task, a participant is asked to remember the word list *car-brick-glasses-mouse*. Positional models, on the one hand, assume that this involves building associations between a positional code and the item itself (e.g., Burgess & Hitch, 2006). That is, the associations *car -[first item], brick -[second item], glasses -[third item]* and *mouse -[fourth item]* would be stored in the case above. To refer to an association between a positional code and an item, we will use the term *serial position-item association*. Inter-item (chaining) theories, on the other hand, assume that sequential learning involves establishing associations between two successive items, such as *car-brick, brick-glasses*, and *glasses-mouse*. These associations are stronger in the forward direction than in the backward direction, in that the activation of brick would lead to the activation of glasses and so forth. These associations will be termed *item-item* or *inter-item associations* in the remainder of the article. Contemporary versions of such models are far more sophisticated than such simple descriptions and often involve a mathematical formulation. For the sake of brevity, however, we will not discuss these details here (for a review, see Houghton & Hartley, 1995).

For the current study, it is crucial to understand in which situations the two classes of theories differ. The most important difference between the two theories regards the role of the preceding item/action for the retrieval of the next. Because from a chaining perspective memory retrieval works via pairwise associations, encountering the (or at least one of the) preceding item(s) is a necessary precondition for retrieval. Serial position theories, in contrast, stress the role of the position an item occupies within a sequence. In its most stringent form, a serial position approach therefore predicts the preceding item to play no role. Rather, serial position serves as a retrieval cue for each item. Therefore, after having stored the above list *car-brick-glasses-mouse*, a serial position theory would predict a performance advantage in storing and performing a different list with one item from the original list that occupies the same serial position, such as *screen-bottle-glasses-photo*. As *glasses* still is the third word of the list, the learned [*third item*] -*glasses* association fosters the retrieval of the item. A chaining model predicts an advantage for a different kind of derived list, in which relations between serial positions and items are changed, but item-item transitions are (partly) retained, such as *brick-glasses-mouse-car*. Here, a specific advantage for *glasses* would be expected because the learned *brick-glasses* association could be reused.

It is important to note that the “problem of serial order” described above is by itself not confined to any particular memory structure, and accordingly it has been a topic of investigation in a variety of research contexts. Interestingly, however, it has been noted that the developments in different research contexts have often mirrored each other, such as in the animal and verbal learning literature (Burns & Dunkman, 2000). In particular, we believe that the questions discussed above are also highly relevant for implicit learning. Two observations motivate this belief: First, serial learning tasks are very prevalent in the implicit learning literature (such as the serial reaction time task [SRT task]; Nissen & Bullemer, 1987). Second, some research has already offered a link between implicit learning and working memory. Frensch and Miner (1994), for instance, suggested a relation between working memory function/capacity and implicit learning (but see Stadler, 1995). Furthermore, Stadler (1993) showed some parallels between implicit learning and the Hebb-learning task (a task developed in the verbal learning literature with the key feature that the same lists are repeatedly presented and thus repeatedly stored in working memory with long term consequences). This is relevant here because in working memory research, the importance of serial position cues and inter-item associations has been the object of many investigations. Against this background, it seems surprising that the central question about the functional stimulus/cue has not been targeted in implicit sequence learning research. Given these observations, as well as our own previous results (Schuck, Gaschler, Keisler, & Frensch, 2011), we believe that the study of item-item and position-item associations in an implicit serial learning paradigm is a valuable goal. In our recent study (Schuck et al., 2011), we already started to shed light on this topic. We reported that implicit knowledge of sequences includes associations between an action and the position, which the action occupies within the sequence. Moreover, we showed that these position-item associations are not the only form of implicit sequence knowledge, as inter-item associations have also been acquired.

While we believe that it is difficult to directly draw conclusions about implicit memory representations of sequential structures from studies in other fields, we acknowledge that evidence for list position-item associations and item-item associations has already been reported in different research contexts. In the case of working memory, for instance, many researchers assume that serial position effects (attributed to position-item associations) rely on mechanisms that are unlikely to play a role in implicit learning. The primacy and recency effects in immediate serial recall, for example, have been attributed to different memory traces, with the latter involving a *verbal* short term store (for a discussion, see Wickelgren, 1973). Hence, we believe that while there are reasons to look for representations of serial order that originate in working memory research, the assumption that they are the same is not warranted. Rather, the question of whether serial position effects can be found in implicit learning therefore becomes all the more interesting. In a few instances, other researchers have also come to similar conclusions (Gershberg & Shimamura, 1994; Mayr, 2009; Raanaas & Magnussen, 2006a, 2006b).

In this article, we studied the acquisition of item-item and serial position-item associations. Specifically, our main interest was in the time course with which these two forms of sequence representations develop and affect performance in an implicit learning task. The work builds on our previous findings (Schuck et al., 2011) that these two representations can be empirically disentangled. Over the course of the present learning situation, we used transfer list techniques to repeatedly estimate the degree to which item-item associations and position-item associations had been formed. These isolated effects can be contrasted with a standard condition in which participants can use both item-item and position-item associations simultaneously.

## Method

### Participants, stimuli, and task

Thirty-one students from Humboldt University Berlin participated for course credits. All participants had normal or corrected to normal vision. Five participants were excluded because they missed at least one session. Another five participants were excluded because they expressed significant amounts of explicit verbal knowledge (see below). The remaining 21 participants (three male, 18 female) had a mean age of 22.1 years (**SD** = 3.7).

**Figure 1.**
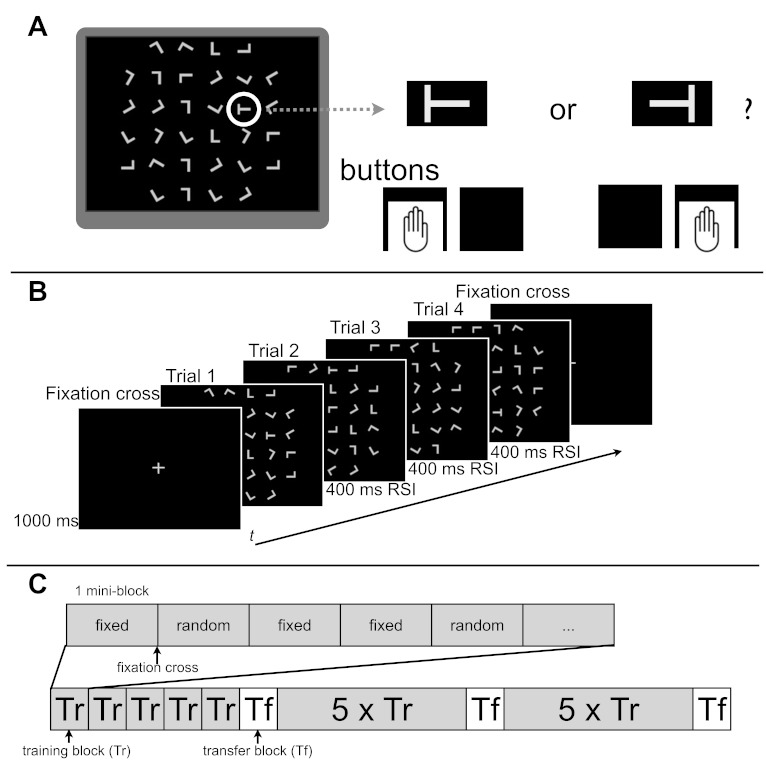
Structure of one trial (Panel A), one mini block (Panel B), and one session (Panel C). A: An example of one trial is shown. In each trial, participants had to search for a tilted *T* among rotated Ls and press a button that corresponded to the tilt of the *T* (left or right). Please note that the target is encircled only for purposes of illustration; during the experiment there was no circle around the target. B: After each fourth trial, a fixation cross appeared on the screen and stayed on for 1,000 ms. The regular response-stimulus-interval (RSI) was 400 ms. C: In each session, five learning blocks were followed by one transfer block. In each learning block, fixed sequence and random sequence mini blocks appeared in random order. For details on the structure of the transfer blocks see text, see Figure 2 and Table 1.

Experiments were programmed in Delphi, using a DirectX component to obtain accurate reaction time (RT) recordings and run on IBM compatible computers with 17-inch screens attached. The [*x*] and [,] keys on a QWERTZ keyboard were assigned to the left and right index fingers, respectively. A *T* and rotated *Ls* (same size) were used as stimuli. They were presented at 32 different locations on a 6 × 6 (minus 4 because the corners were left empty) quadratic grid matrix on the display screen (see Figure 1, Panel A). Each cell in the grid measured 96 by 96 pixels at a screen resolution of 1024 × 768 pixels. Participants were seated about 60 cm from the screen, with the result that each rotated *T* or *L* covered a visual angle of about 3.01°.

The experimental task was identical to the one used previously by Schuck et al. (2011). Participants were asked to complete a visual search task in which the tilt of the target letter determined the button press. A *T* served as the target and rotated *Ls* (same size) as distracters. In each trial, the target appeared on the screen at one of 32 possible locations and distracters occupied the remaining 31 locations. If the *T* was tilted to the left, participants were to depress the left key; the right key was to be depressed for a *T* tilted to the right. Errors were followed by a tone. The regular response-stimulus interval (RSI) was 400 ms. Figure 1 (Panel A) illustrates the setup of a trial.

### Design and procedure

A fixation cross appeared after each fourth trial for 1,000 ms, dividing all trials into mini blocks of 4 trials each. These mini blocks served as the basic building blocks of the experiment (Figure 1, Panels B and C). Depending on the condition of the mini block (see below) the sequences of successive target screen locations within that mini block followed different sequential regularities. Thus, in the current experiment a *sequence* refers to four target screen locations within a mini block. A target screen location serves the role of an *item*, and we will use this terminology when we link our results to other serial learning research. Twenty-four mini blocks constituted a block (96 trials). After each block, participants received feedback about their performance (mean RT) and had a chance to take a short break. Each session consisted of 18 blocks. Overall three sessions (54 blocks á 96 trials) were administered within one week. Two consecutive sessions were separated by two days. In each session, participants were asked to perform the same task without any apparent changes. Figure 1 (Panel C) illustrates the structure of one session. The experiment spanned three sessions with three test phases each. The design allowed us to explore the dynamics of the acquisition and application of different forms of sequence knowledge.

Each block fell into one of two categories: training or transfer block. The statistical properties of the sequences that comprised the mini blocks were determined by the condition of a particular block. The different statistical properties of sequences were tailored to answer the above outlined questions about serial position and inter-item associations. Below we will describe the different types of blocks.

#### Training Blocks

**Figure 2.**
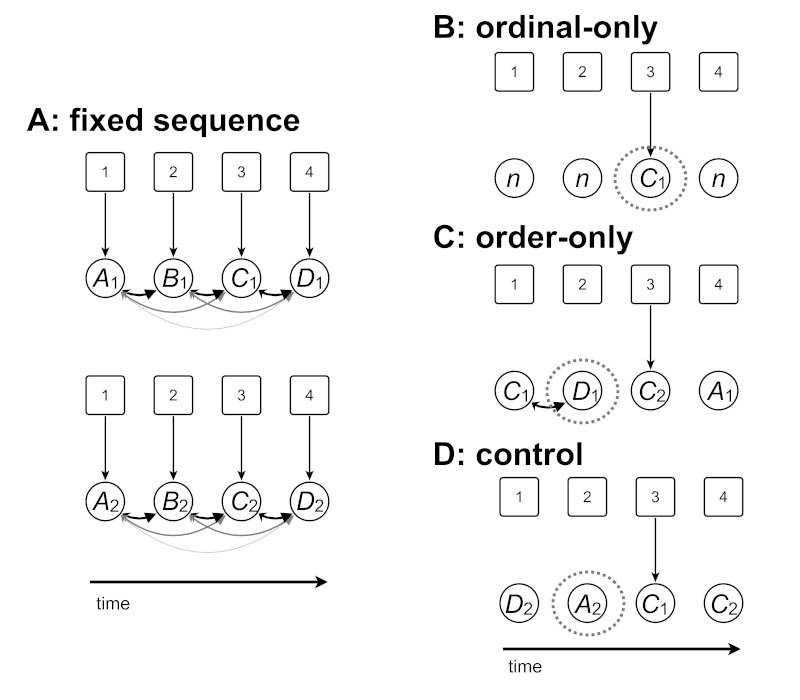
Schematic illustration of memory structures of fixed sequences (Panel A) and derived transfer sequences (Panels B-D). In all cases, encircled letters correspond to elements of a sequence, with sequential presentation going from left to right. The boxed numbers above the sequence elements indicate representations of the respective serial positions. Arrows correspond to associations. A: In our view, sequence learning results in the formation of item-item as well as of position-item associations. The former are indicated by the round arrows between sequence elements, the latter by the straight arrows between the serial positions and the sequence elements. In the learning blocks, two repeated fixed sequences could be learned. It is important to note that participants learned two different sequences, *A_1_-B_1_-C_1_-D_1_* and *A_2_-B_2_-C_2_-D_2_*. The italic letters indicate a sequence element and the indices the sequence identity. Therefore, *A_2_* corresponds to a different target screen location than *A_1_*, etc. B: To test for position-item associations, the ordinal-only sequences feature trials that have not been used during learning (indicated as *n*), as well as test trials where a target screen location from one of the learned sequences occupied the same serial position, *n-n-C_1_-n*. (Element *C_1_*, now being the third element in the sequence, as in the upper part for Panel A.) C: Only item-item association information is available. In this case, an order-only trial needs to be preceded by the same sequence element as it is during the learning phase. For example, in the sequence *C_1_-D_1_-C_2_-A_1_*, element *D_1_* is preceded by element C_1_ as during the learning phase (importantly, *C_1_* and *D1* both are from the same, but *C_2_* is from a different sequence, as mirrored by the indices), so the reaction time (RT) during the trial with element *D_1_* is considered (see Panel C). D: Situations where no associative knowledge could be used for prediction/retrieval facilitation. In this case *A_2_* is now preceded by *D_2_*, unlike in the learning phase. Hence the RTs in the trial where the target appeared at screen location *A_2_* are considered. Please note that unlike in the examples, the test item appeared at all possible serial positions, not only at the third serial position. Analyzing trials where two target screen locations appear in the learned order at the wrong serial position can provide insights into item-item associations.

In training blocks, item sequences within mini blocks were either fixed or random (see Figure 1, Panel C). Two sets of four items each were used in the fixed sequences; the four items (i.e., target locations) always occurred in the exact same sequence in each mini block. Consequently, the fixed sequences exhibit sequential structure in two ways: first, the transition probability between two target screen locations was first order deterministic. Second, the sequences feature deterministic contingencies between serial positions and target screen locations (a certain target screen location was always at the same serial position within a sequence). The two properties of the fixed sequences are schematically illustrated in Figure 2 (Panel A).

Two different sets of four items each were used in the *random sequences*; they were shown in an order that changed between mini blocks (e.g., *K-L-M-N* in one mini block and *N-K-L-M* in another mini block, with letters indicating target screen locations). Accordingly, for each participant the same target screen locations were used throughout and the order was the result of a random draw (without replacement). Hence, neither transition probabilities nor position-screen location contingencies were deterministic in a random sequence. Table 1 provides examples of fixed and random sequences.

**Table 1.** Schematic Examples of Training and Transfer Sequences.

Fixed sequences	A – B – C –D
	a – b – c – d
Random sequences	K – G – M – N
	M – K – G – N
	G – N – K – M
	L – P – F – H
	H – L – P – F
	P – F – H – L
Order-only	B – **C** – A – d
	A – d – a – **b**
	a – C – **D** – B
Ordinal-only	Q – **B** – X – Z
	**a** – R – U – T	
	V – Y – **c** – W

*Note*. Letters indicate different target screen locations. In the order-only and ordinal-only rows, the bold letters indicate the trials of which reaction times are analyzed. In the order-only sequences, the bold letters are always preceded by the respective previous items from the same sequence (capital *C* is preceded by capital *B*), while the serial position is incorrect (*C* appears in the second serial position). Compare Panel C of Figure 2. In the ordinal only condition, the single target screen locations from the fixed sequence condition are embedded in previously unused target screen locations (notated as n in Figure 2, Panel B, here new items are *R*, *Q*, *T*, *U*, *V*, *W*, *Z*). At the same time, the fixed sequence target screen locations appear at their correct serial position (*B* is in the second serial position in the first example, a is in the first serial position in second example, etc.).

In all sequences the tilts and thus the required manual reaction (left vs. right) were semi-randomly determined (ensuring the same number of right and left responses in each block). The assignment of items to the fixed sequences or to the random sequences was counterbalanced between participants, preventing differences in salient screen locations or mean distance from the fixation cross to be confounded with the reported RT differences. The sequences within mini blocks were constructed such that two consecutive items could not appear in neighboring locations on the screen. Each training block consisted of 24 mini blocks of which 16 contained one of the two fixed sequences (i.e., eight mini blocks with Sequence 1 and eight with Sequence 2). The remaining eight mini blocks contained either of the two sets of the random sequence items in random order in equal frequency. Within one session, 15 training blocks were used. Thus, within one session all participants responded to each of the two fixed sequences 120 times during the training phase and to each of the random sequences 60 times. All mini blocks appeared in pseudo-random order, excluding the possibility of more than three consecutive mini blocks in the same condition. Only half of all possible 32 items were used during training, leaving 16 unused items for the construction of the transfer sequences.

#### Transfer Blocks

Each session contained three evenly spaced transfer blocks (blocks 6, 12, 18). In the transfer blocks we tested for implicit learning of two different types of information (i.e., item-item and position-item associations). Three different types of transfer conditions were applied in counterbalanced order and targeted the two different types of sequence knowledge.

Our general approach was to use the method of derived lists (Ebbinghaus, 1885/1992; see also Chen, Swartz, & Terrace, 1997). The idea of derived lists is to use transfer lists that share some features with previously learned lists, but not others (i.e., they are derived from the originally learned lists). As mentioned in the Introduction, having learned a certain list should have effects on new lists. Chaining and serial position theories make different predictions for such lists. Our transfer sequences were constructed to tap exactly into these differences. It was varied whether in a transfer list (a) the serial position of a target screen location, (b) the preceding target screen location, or (c) none of the two was kept (so that nothing was identical to the fixed sequences from the learning phase other than the item identity). Performance in these transfer lists can be used to investigate the acquisition of (a) serial position and (b) chaining information, respectively, and to compare it to a baseline (c). In each case, the new transfer sequences consisted of four trials with intervening fixation crosses (as in the learning blocks). The fixed sequence items we reused in the transfer lists were drawn such that all sequence items were used equally often in the transfer sequences. Figure 2 (Panels B, C, and D) illustrates the logic of the transfer sequence construction and analysis and Table 1 provides examples.

The *ordinal-only* transfer was constructed to test for serial position knowledge of the trained sequences. Therefore, the transfer sequences had two properties: First, one item was at the same serial position as during learning (the ordinal-only trial, e.g., the third target screen location within a fixed sequence mini block was now also the third target screen location). Second, in order to exclude chaining information from interfering, the preceding item had to be different from the originally learned list. Hence, in the remaining three trials of these sequences, the target appeared at previously unused target screen locations (new-location trials, in Figure 2, Panel B, denoted as *n*). The construction of such sequences is illustrated in Panel B of Figure 2 and in Table 1. As the figure illustrates, only serial position theories would expect a specific advantage of ordinal-only trials in such sequences. Hence, any RT advantage of an ordinal-only target location relative to a new-location trial provides evidence of the acquisition of serial position information. The *ordinal-only* estimation we will use in the Results section refers to the difference between new-location trials and ordinal-only trials in mini blocks of the ordinal-only condition. This means that we computed the difference between the above described trials and trials in which the target appeared at previously unused screen locations. Please note that we used previously unused target screen locations in order to avoid interference from inter-item associations. In this manner, it is an important improvement over previous attempts to measure serial position-item associations. If another item from a previously learned sequence preceded the trial we used here to estimate serial position knowledge, then this item would lead to the activation of the item that was next in the original sequence via inter-item associations and therefore interfere with the search for the target (cf. Ebenholtz, 1963).

In order to estimate the acquisition of chaining information, we used *order-only* transfer sequences. These sequences had properties complementary to the ordinal-only sequences: The preceding item must be the same as in the learned list (so that a learned inter-item association leads to the retrieval of the correct item), but the correct pair has to appear at the wrong serial position, in order to prevent the assistance of serial position information. Figure 2 (Panel C) illustrates these principles. As can be seen, target screen locations from the fixed sequences were used to construct the sequences (see Panel C of Figure 2 and the examples in Table 1). In these transfer sequences, we consider trials where in the preceding trial the target was at the same location as in the learned sequence, while the considered trial itself is at the wrong serial position. In this situation, inter-item but not serial position knowledge associations can lead to faster RTs. Hence, we computed the difference between the above described trials and trials where the target appeared at previously unused screen locations, that is, new-location trials in the ordinal-only condition. Table 1 provides examples of order-only and ordinal-only sequences.

Finally, we considered trials where two consecutive fixed sequence target screen locations neither had the same order as before nor appeared at their correct serial position. Hence neither a chaining nor a serial order account would predict an RT advantage (Figure 2, Panel D). Consequently, we took these RTs as a control, that is, a no association condition (*control trials*).

In each transfer block, eight mini blocks contained sequences with ordinal-only trials, eight mini blocks sequences with order-only trials, and eight mini blocks random transfer trials.^1^ Control trials could be extracted from mini blocks containing order-only trials (see Figure 2; sequences C and D are equivalent in the sense that they were constructed of one target screen location from the fixed sequence condition at the correct serial position and three target screen locations from the other fixed sequence that were at the wrong serial positions). Accordingly, all transfer blocks contained all transfer conditions.

#### Explicit knowledge assessment

Because it is important to establish that the learning phenomenon we study here is implicit in its nature, we conducted assessments of verbal knowledge after the main experiment (i.e., after Session 3). Consequently, we excluded all participants exceeding a certain threshold of verbal knowledge from analysis. To do so, the instructor provided each participant with a sheet containing two grids representing the possible locations on the screen (a 6 × 6 square with omitted corners). Subjects then were told about the existence of two fixed regular sequences in the experiment and were asked to try to recall at which locations and in which order the targets appeared most often during the experiment. The cells in the grid indicated the different locations on the screen and had to be marked with the numbers 1 to 4 to indicate the order of target locations. Participants were also asked to indicate which screen locations had been occupied by the random sequences and at which locations the target only very rarely had appeared.

## Results

All analyses were conducted using R (R Development Core Team, 2010). For all analyses conducted with RTs in the following sections, erroneous responses and responses following errors were excluded. To reduce the influence of outliers, analyses were conducted based on the median RT for each participant in each of the factor cells (Luce, 1991) that constituted the analysis. Thus, unless otherwise noted, analyses were based on the individual median RTs per block. The p-values accompanying correlations are according estimations as implemented in the stats package in R (R Development Core Team, 2010).

### Fixed and random sequences

**Figure 3.**
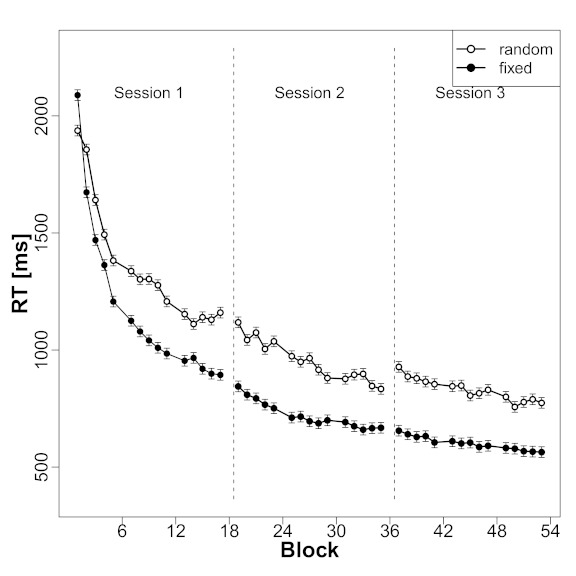
Development of reaction times (RTs) during the learning phase. The figure shows mean RTs from the fixed sequence (solid circles) and random (empty circles) conditions as a function of block. Vertical dashed lines indicate the beginning/end of a session (about 48 hr without training). Bars indicate standard errors for within-subject designs (based on the interaction effect, see Loftus & Masson, 1994).

To evaluate the development of overall sequence knowledge, we considered trials from the training blocks for the fixed and random sequences. Figure 3 shows the mean RTs for the two conditions as a function of block. As can be seen, reactions in both kinds of sequences speeded up over time. At the same time, RTs in the fixed sequences decreased at a faster pace than RTs in the random sequences. Whereas participants responded slower to fixed sequence trials than to random trials in the first block (mean difference: 151 ms), this difference was already drastically reversed in the second block (183 ms). Furthermore, the difference continued to increase between the first and the second session, with mean differences in Sessions 1, 2, and 3 being 184 ms, 232 ms, and 229 ms, respectively. To support these impressions, we computed a two-way repeated measures ANOVA with Block (running block count of training blocks over all sessions, 45 levels) and Sequence Type (two levels: fixed sequence vs. random) as factors.

The overall speed-up of RTs regardless of sequence type is reflected in a main effect of block, *F*(44, 880) = 141.91, *p* < .01. The observation that RTs in the fixed sequences were generally faster than RTs in the random sequences is supported by a main effect of sequence kind, *F*(1, 20) = 35.61, *p* < .01. Importantly, the fact that the difference between fixed and random sequences grew larger over the course of time is captured by an interaction between Block and Sequence Type, *F*(44, 880) = 4.28, *p* < .01. This latter result strongly suggests that with training, participants acquired knowledge about the fixed sequence that speeded up their reactions in trials where successive target locations followed a fixed sequential regularity. Figure 3 reveals that this interaction is driven by the changes taking place in the first and second session.

### Sequence transfer conditions

**Figure 4.**
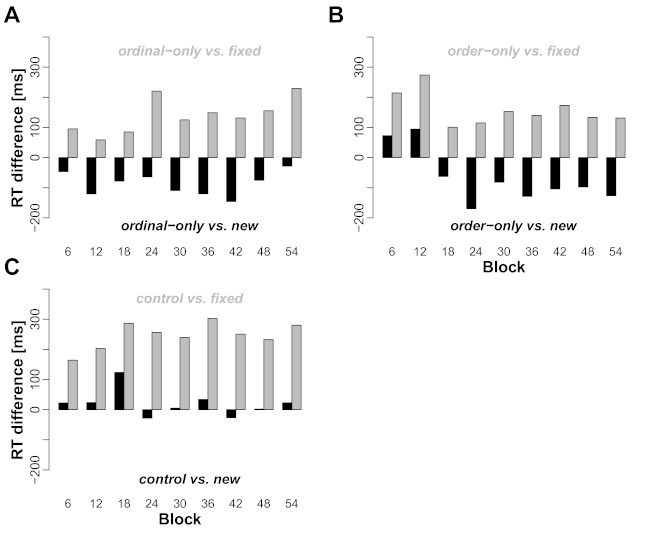
Reaction times (RTs) in different transfer sequence conditions relative to RTs in fixed sequences and new-location trials. A: For each transfer block, the RTRT difference between test trials in new-transfer sequences (ordinal-only) and fixed sequences (gray bars) and new-location trials (black bars) are shown. Panels B and C also show the two respective differences, where in Panel B order-only trials are taken as reference and in Panel C control trials. For further descriptions, see text.

The above analysis suggests that participants acquired sequence knowledge during the training blocks. In a next step, we analyzed the transfer blocks in order to decompose overall sequence knowledge into its constituents. Our main goal was to separately estimate the strength of item-item and position-item associations in isolation and compare these with the combined use of the two associations. Since we have multiple assessments of the two types of associations over time, we can investigate possible training related changes of the relative contributions of these associations. As explained above, we used the method of derived lists. This method allowed us to evaluate performance in sequences where (a) only the serial position structure (ordinal-only), (b) only the order structure (which item follows which, order-only), or (c) neither one was maintained relative to the fixed sequences (control). While in the former cases, the screen location of the upcoming target could be predicted based on one of two kinds of sequence knowledge, no sequence information could be applied for speeding up the search process in the control condition. In the Methods section, we explained the details of these conditions. As mentioned, RTs from each of these three cases were compared to RTs from new-location trials. This difference reflects a “pure measure” of the respective knowledge sources, as it is assumed that no or very little knowledge is available about the new sequence items. In addition, we also compared the RTs in the three transfer conditions to RTs in fixed sequences. This difference is indicative of the relative contribution of the respective knowledge source to the performance in a standard sequence, where both types of information are available. Figure 4 shows the two difference scores (black and grey bars, respectively) for each of the transfer conditions (Panels A, B, and C, respectively).

### Position-item associations

Figure 4 (Panel A) shows the RT differences of ordinal-only trials to unused target screen locations (black bars) and to trials from the fixed sequence condition (gray bars) from the preceding block. A positive difference indicates that ordinal-only trials are slower than the respective comparison, and vice versa. It can be seen that (a) ordinal-only trials are consistently slower than fixed sequence trials and that (b) ordinal-only trials are consistently faster than new-location trials. To test observation (a), we computed a two-way within subjects ANOVA with factors Session (three levels) and Condition (two levels, ordinal-only vs. fixed sequence trials). The observation of slower RTs in ordinal-only trials than in fixed sequence trials was confirmed by a main effect of Condition, *F*(1, 20) = 18.63, *p* < .01. As expected, we found also a main effect of session, *F*(2, 40) = 129.68, *p* < .01. The interaction of Condition and Session was at the margin of significance, *F*(2, 40) = 3.03, *p* =.06. This interaction was driven by an increasing difference, with the mean difference for the sessions being 80 ms, 165 ms, and 172 ms. A linear regression of session on the difference score of fixed sequence versus ordinal-only trials confirmed that session significantly predicted the RT difference, ß = 0.15, *t*(187) = 2.08, *p* < .05, *R*^2^ = 0.02. Finally, we performed a similar two-way ANOVA comparing ordinal-only with new-location trials (i.e., including factors session and condition). The observation of faster RTs in ordinal-only than in new-location trials was confirmed by a main effect of condition, *F*(1, 20) = 15.20, *p* < .01 (mean difference: 88 ms). The main effect of session was also significant, *F*(2, 40) = 109.21, *p* < .01. No interaction between Session and Condition was found, *F*(2, 40) < 1.

To summarize, we found large RT advantages that can be taken to reflect serial position-item associations alone (a main effect when ordinal-only trials are compared to new-location trials). We also found RT disadvantages when ordinal-only trials were compared to RTs from a fixed sequences condition, indicating that the serial position-item associations are not sufficient to explain the entire RT advantages in intact sequences. Moreover, we did not find a Session × Condition interaction when order-only trials are compared to new-location trials (the measure of serial position - item associations), indicating that the associations are already learned very early in training. In contrast, however, we found that these associations can increasingly explain less of the RT advantage one finds when intact sequences are considered (i.e., we found a linear increase in the difference between ordinal-only and fixed sequence trials).

### Item-item associations

As explained above and as shown in Figure 2 (Panel C), RTs in the order-only condition are indicative of item-item associations. Specifically, we considered trials where two succeeding target screen locations were in accordance with acquired item-item associations but appeared at the wrong ordinal position. As in the analysis of position-item associations, these trials were contrasted with new-location trials from the ordinal-only condition and with trials from the fixed sequence condition.

The respective RT differences can be seen in Panel B of Figure 4. Overall, RTs in order-only trials were slower than in fixed sequence trials, but faster than in new-location trials. The observations were tested in the same manner as before with repeated measures ANOVAs. It can be seen that there was a marked difference between RTs in order-only and fixed sequence trials (gray bars). Also, a difference between order-only trials and new-location trials could be observed (black bars). The first difference was confirmed as statistically significant: We found a main effect of condition in the comparisons between order-only versus fixed sequence trials, *F*(1, 20) = 24.33, *p* < .01, whereas the second observation was supported by a marginal main effect of condition for the order-only versus new-location comparison, *F*(1, 20) = 3.62, *p* =.07 (mean difference: 67 ms). In addition, in the ANOVA comparing fixed sequence and order-only RTs, we obtained a main effect of session, *F*(2, 40) = 153.50, *p* < .01, but no interaction between Session and Condition, *F*(2, 40) < 1. In contrast, for the second ANOVA in which the conditions order-only and new-location were compared, we found both a main effect of session, *F*(2, 40) = 136.47, *p* < .01 as well as an interaction *F*(2, 40) = 5.00, *p* < .05. Figure 4 reveals that in the first two blocks, the difference between order-only and new trials is positive in the first two blocks and negative in the remaining blocks, causing the interaction and diminishing the main effect of condition. Again, we investigated the possibility of a linear trend by submitting the individual blockwise RT differences to a regression with factor session. This analysis indeed supports such a relationship, ß = 0.17, *t*(187) = 2.36, *p* < .05, R2 = 0.03.

To summarize, we found RT evidence for item-item associations that - unlike the evidence for serial-position associations - emerged during the first session and increased over time. In contrast, the difference to the RTs in fixed sequence was consistent throughout all sessions and did not show an interaction with training session.

### Control

Finally, we considered trials where neither item-item nor serial position-item associations could be used to predict the screen location of an upcoming target. This analysis served as an important control for our transfer-list approach. As can be seen in Figure 4 (Panel C), these trials were considerably slower than fixed sequence trials but did not differ reliably from the new-location condition. Respective ANOVAs again confirmed these observations. The ANOVA contrasting fixed sequence trials with the control trials showed main effects of condition, *F*(1, 20) = 41.89, *p* < .01, and session, *F*(2, 40) = 126.16, *p* < .01, but no interaction between Condition and Session, *F*(2, 40) < 1. In contrast, an ANOVA with the control trials and the new location trials showed no main effect of condition, *F*(1, 20) < 1 (mean difference: 20 ms), or interaction, *F*(2, 40) = 1.35, *p* =.27. As expected, we found a main effect of session, *F*(2, 40) = 103.78, *p* < .01. This suggests that targets that were supported neither by item-item nor by serial position-item associations were located just as slowly as targets not used in the learning blocks. Thus, apparently there was no advantage of targets used in the learning blocks over targets not used in the learning blocks that was independent of two forms of sequence knowledge. Because the trials we use here were trials that appeared in the immediate environment (within the same mini blocks) as the crucial transfer conditions we considered above, the reported pattern supports the notion that the transfer effects are specific to differences in sequential structure.

### Independence of item-item and position-item associations: Race model test

In the previous section, we observed that RTs for trials in fixed sequences are shorter than RTs for order-only and ordinal-only trials (which still show an advantage over randomly ordered or novel targets). The fixed sequence trials correspond to a situation in which item-item as well as position-item associations can be used to predict the next target screen location. Larger RT advantages in a situation in which two forms rather than one form of sequence knowledge can be applied could potentially be rooted in two different forms of expression of these knowledge sources. According to the first option, two forms of evidence accumulate in independent pools. Item-item as well as position-item associations influence the search process independently of one another. As detailed below, a race-metaphor has been proposed to capture the essence of this account. When both rather than just one of the knowledge sources can be applied, two memory sources are racing for retrieval. In this scenario, the first source that is retrieved determines behavior and in consequence a purely statistical facilitation effect can be observed: the fastest of two (or many) sources in a race can be expected to be faster than the fastest of one source (or few). Even though the racers run entirely independently of one another, a faster response can be expected in cases where two forms of sequence knowledge are applicable. Therefore, even though the two memory traces are independent of one another, an over-additive effect can be expected. According to the second account, two types of evidence accumulate into a single pool. Item-item as well as position-item associations jointly determine the search process. The gain based on multiple as compared to single knowledge sources can be expected to be larger than in the case of independent accumulation of evidence in separate pools; as a joint accumulation based on two knowledge sources can cross the threshold to drive the search process faster than accumulation based on a single knowledge source could.

The above distinction has been discussed and modeled in the literature on the redundant stimulus effect (RSE; e.g., Miller & Ulrich, 2003) and pinned down to test for violations of the race model inequality (Ulrich, Miller, & Schröter, 2007). An analysis of RT distributions can help to differentiate between the two different interpretations of RT advantages in a situation that allows for two rather than for one source to influence a response process. The goal of this analysis is to determine whether the fast RTs in trials with multiple knowledge sources are even faster than could be expected based on statistical facilitation. By extension, this analysis then allows us to draw conclusions on the independence of the two knowledge sources we investigate in the present article.

The search processes in order-only and ordinal-only trials are supported by one kind of sequence knowledge each. The corresponding single source RTs will be called *RT(order)* and *RT(ordinal)*, respectively. Correspondingly, RTs to target screen locations in the fixed sequences will be considered as the combined condition, *RT(fixed sequence)*. Here, both kinds of sequence knowledge could support the search process. In this context, it seems noteworthy that the individual overall RT-based estimations of serial position-item and inter-item association strength, correlate highly with each other, *r* =.51, *t*(19) = 2.65, *p* < .05, as well as with the difference between fixed and random sequences, *r* =.76 and *r* =.54, respectively, both *ps* < .05. The same is true when the combined (order-only plus ordinal-only) RT scores are correlated with the fixed versus random sequence difference, *r* =.71, *t*(19) = 4.46, *p* < .01 (all correlations are Pearson product moment correlations).

Moreover, it seems noteworthy that we found that the mean RT advantage for RT(order) over new-location trials was 67 ms, and the corresponding advantage for the RT(ordinal) trials was 88 ms. In contrast, the mean RT advantage of RT(fixed) over random sequence trials in the first session was already 187 ms; well above the (additively) combined effect of both single memory process conditions (67 + 88 = 155 ms). The mean overall difference between random and fixed sequence was 221 ms and indeed marginally different from the combined effect of 155 ms, *t*(20) = 1.89, *p* = .07. Hence, while there is a strong relation between the two contributions from the two association forms to the performance in a standard sequence, our data also give rise to doubts whether the contributions from both associations are additive (i.e., independent).

While over-additivity in general seems to point toward non-independence, statistical considerations about summation of probabilities need to be taken into account. Specifically, because the RT that is recorded in each trial reflects only the faster of two processes, the result will be subject to a statistical facilitation effect. As we explained above, this statistical facilitation comes about because, having two independent distributions, drawing from the two distributions but considering only the minimum of the obtained sample leads to a lower estimate of the minimum than the estimate of that minimum one would obtain from a combined distribution. The theory of race models takes advantage of this fact to make a prediction at the level of cumulated density functions (CDFs) of the RTs. According to this prediction, independence of the two processes cannot be rejected as long as the race model inequality holds: *F_z_(t)* <= *F_x_(t)* + *F_z_(t)* (1)

where *F_x_*, *F_y_* are the CDFs of the single stimulus conditions with features *x*, *y*; and *F_z_* is the CDF for the combined condition *x* and *y*. Conversely, a violation of the race model inequality would speak for a joint rather than independent influence of the two forms of sequence knowledge on the search.

**Figure 5.**
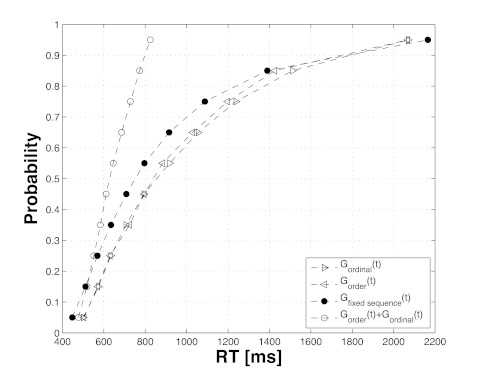
Estimated cumulative density functions for order, ordinal, and fixed sequence trials. The figure shows the 10 estimated percentile points for each of the four functions of interest: *G*_order_, *G*_ordinal_, *G*_fixed sequence_. The figure also displays the calculated *G* estimates of *G*_order_ + *G*_ordinal_, which is central to assess the validity of the race model inequality. RT = reaction time.

We applied this prediction to the RT distributions we obtained for RT(order), RT(ordinal), and RT(fixed sequence) to obtain estimates of the CDFs, *G*_order_, *G*_ordinal_, and *G*_fixed sequence_. The CDFs were calculated for each participant separately. The procedure is detailed in Ulrich et al. (2007). The corresponding mean CDFs are shown in Figure 5. As can be seen, the observed CDF for RT(order + ordinal), *G*_order_+ *G*_ordinal_, lies in most cases well above the calculated CDF for RT(fixed sequence), *G*_fixed_ sequence. However, for the very fast RTs reflected in the first percentile, the *G*_order_+ *G*_ordinal_ CDF lies (empty circles) below the *G*_fixed sequence_ (solid circles) and thus seems to indicate a violation of the race model inequality (see Formula 1). The RTs calculated for the first percentile of the fixed sequence are faster than the RTs estimated for the first percentile of the joint distribution of the order and the ordinal condition. A corresponding t-test, comparing the mean RTs in the first percentile of the estimated CDFs for the order+ordinal and the fixed sequence conditions marked this difference as significant, *t*(20) = 2.19, *p* =.02 (paired t-test, one-tailed, without Bonferroni correction). The respective means are 447 and 477 ms. One should keep in mind that a Bonferroni correction would be too conservative as violations of the race model inequality can only occur in the very first percentiles, but some correction would usually be required (for simulations, cf. e.g., Kiesel, Miller, Ulrich, 2007). Kiesel and colleagues suggested adjusting the *p*-values for a restricted range of percentiles where the violations are usually found (10-25%). Because we used a different segmentation into percentiles here in our example, this corresponds to the range of 5-20% and involves two comparisons (5% and 15%). The *p*-value we reported above (.02) would survive such a correction.

Thus, the observed RT distributions for the different conditions support the view that item-item and position-item associations are non-independent processes (i.e., they influence RT jointly rather than independently of one another). Despite this analysis, we acknowledge that this conclusion is of limited certainty. This limitation rests on the fact that the RTs in the single conditions we used to estimate an order-only and ordinal-only situation are very likely to reflect more than just the respective single process condition. Specifically, as discussed already above, the different preceding trials in the different sequences from which we extracted the RTs also probably have effects on the RTs we observed.

### Explicit knowledge

The amount of explicit knowledge was analyzed using a two-step process: First, the overlap of each participant’s report was quantified by comparing it to the appropriate probability distribution for the case of guessing. This yielded a score that reflected the probability that a participant would get the observed amount of overlap with the true sequence if she/he was guessing. If this probability was smaller than 5%, the participant was excluded from all analyses. Secondly, we correlated the individual probability scores to the amount of learning as reflected by the RTs in the training blocks. Due to human error, two reports were lost and therefore excluded. All reported rs are Spearman rank correlations and the accompanying *p*-values are according estimations as implemented in the stats package in R (R Development Core Team, 2010).

To calculate the probability scores, we considered the fixed and random sequences the participants generated in the interview. In the first case, the number of hits was counted. Only a correct screen location at the correct ordinal position was considered a hit. The probability of obtaining the different numbers of hits by guessing was estimated by generating 107 random sequences of the 32 possible numbers and counting the number of events where 0, 1, 2, 3, or 4 sequence elements corresponded to a randomly selected sequence in that order (cf. Rünger & Frensch, 2008). For each participant, the two probabilities from the two generated fixed sequences were averaged. In the same manner, we assigned probability scores for the generated random sequences. There, however, the order of report did not matter. Consequently, all correct target screen locations were counted as a hit and the number of hits was transformed into a probability by using a hypergeometric density function. As a result of this analysis, five participants were excluded. The probability scores for random and fixed sequences were correlated (*r* =.37, *p* =.07). The mean combined score of the remaining participants was low (*M* =0.70, *SD* = 0.27, with the probability that the reported sequence is random being the unit), and did not correlate with the individual mean RT difference between fixed and random sequences in the last five training blocks, *r* =-.03, *p* =.90. Hence, there seems to be no relation between the extent of explicit knowledge and the extent of sequence knowledge as reflected by RTs.

Finally, using the same procedure as for the random sequences we analyzed the reports of the rarely used locations. We found that four additional participants reported a number of rarely used target locations that is unlikely (< 5%) if they were guessing. Notably, one of these participants actually reported a significantly smaller number of correct locations. We did not exclude participants based on this score for three reasons:

1. In the current study, our main focus was on the implicit learning of sequence knowledge, not frequency knowledge.

2. There were low and non-significant correlations between this score and the random and fixed sequences scores, *r* =.32, *p* =.13, and *r* =.13, *p* =.52, respectively.

3. The score appeared to have no significant relation to the performance in the new-transfer sequences, *r* =.29, *p* =.23.

## Discussion

We propose - in line with research from other serial learning tasks - that in the present task, implicit sequence knowledge may represent (a) transposition probabilities between successive target screen locations, and (b) contingencies between serial positions and target screen locations (e.g., Ebenholtz, 1963, 1966; Young, 1962). We hypothesized that these kinds of information are stored in (a) item-item associations and (b) associations between serial positions and items, respectively. Unlike in many other experiments, we based our analyses on the assumption that both types of associations are actively and simultaneously supporting serial learning. To test our assumption, we administered transfer blocks in regular intervals throughout a prolonged practice phase of a serial reaction time task. In these transfer blocks, the targets appeared in new sequences that were derived from the learned sequences. The analysis of RTs in these sequences then allowed us to test separately if item-item and serial position-item associations had been acquired.

Our main result was that we indeed found evidence for the acquisition of both kinds of associations. Moreover, we obtained two additional results: first, the size of the RT advantage for sequences that allow the use of learned position-item or item-item associations separately was much smaller than the RT advantage for fixed sequences where both associations can be used simultaneously (i.e., we found significant main effects for condition when we compared the ordinal-only and order-only trials with the fixed sequence trials). Also, the combined (additive) effect does not match the RT advantage of a fixed sequence structure. Additionally, the RT distributions we obtained in the order-only, ordinal-only, and fixed sequence conditions violated the race model inequality. Hence, we found some indications that the two types of associations do not work independently when both can be applied.

Second, we found training related changes of the observed associations. Relative to the development of overall sequence knowledge as expressed in the difference between random and fixed sequence trials in the learning blocks, the isolated impact of the two forms of sequence knowledge in test blocks changed differentially with ongoing practice. Performance in the ordinal-only and order-only trials was evaluated relative to the performance in the fixed sequence trials of the previous learning block. Thus, these difference scores reflect the relative contribution of either form of sequence knowledge to overall sequence knowledge at that point in training. We found that this difference was growing with practice for the ordinal-only trials but not for the order-only trials. In addition, we computed a measure of the respective associations by comparing the order-only and ordinal-only trials with trials in which the target appeared at previously unused screen locations. This analysis revealed that whereas the impact of item-item associations on the search process became evident only after the first session and showed a linear increase with practice, the position-item associations did not seem to change with practice. This picture fits very well with the findings we obtained when we compared the transfer condition to the fixed sequence condition: Whereas the serial position-item associations seemed to contribute less and less to the RT advantage for fixed sequences, the strength of item-item associations increased. Taken together, this picture is consistent with the idea that with ongoing practice, item-item associations become relatively more important for the process that leads to the observable RT advantage of a standard fixed sequence over a random control.

Overall, our results are well in line with previous findings in serial learning experiments. In serial recall tasks, for instance, evidence for the use of item-item associations and position-item associations was already reported already very early on by Ebenholtz in 1963. Additionally, however, our results add important new insights to the existing literature: First, an exhaustive formulation of the sequential structure that is learned in implicit serial learning is still missing. Despite existing considerations about various kinds of sequential dependencies (Hoffmann & Koch, 1998), the notion of position-item contingencies has not been taken into account. Our previous study (Schuck et al., 2011) is the first to suggest that this is necessary to fully understand implicit sequence learning.

It is important to discuss the relevance of the present findings for standard SRT experiments. In the present study, the start and end of each sequence was indicated by a fixation cross. This is not the case in typical SRT experiments, where successive trials appear without any segmentation. Thus, one might argue that position-item associations cannot develop in a typical SRT task. However, please note that it is possible that the participants used statistical structures to segment the stream of ongoing trials. Cohen and colleagues, for instance, argued that changes in transposition probabilities that occur at the boundaries between two sequences might be used as anchors for segmentation (Cohen, Ivry, & Keele, 1990; see also Stadler, 1992). This is also supported by research on the learning of word segmentation (Saffran, Newport, & Aslin, 1996). In addition, the task we used here shares features with some published implicit learning experiments. Tunney (2003), for instance, used the words start and end as explicit segmentation cues between sequences generated by an artificial grammar (see also Tamayo & Frensch, 2007; for other sequence learning paradigms that include start cues, see e.g., Perlman & Tzelgov, 2009; Stadler, 1989; Ziessler, 1998). Nevertheless, we acknowledge that the numerically smaller estimation of item-item associations than serial position-item associations (67 ms vs. 88 ms, respectively, difference not significant, *t*(20) < 1) is surprising and might point towards an underestimation of item-item associations as compared to standard designs. One likely contribution to this finding is that whereas the ordinal-only condition is relatively free from interferences (because the preceding trials are new-location trials), this is not the case for the order-only condition. In this case, trials coming before the crucial correct pairwise transition from one location to another might cause interference and hence impair the estimation of item-item associations. In addition, we argue that our study may be informative even for serial learning in explicit tasks. Despite much debate about the functional stimulus in serial learning (Young, 1962; Young, Hackes, & Hicks, 1967), it has not been experimentally tested whether the combination of the two alternatives in the debate on the nature of the representation of serial order, the previous stimulus and the serial position, might serve as functional stimulus when fixed sequences are learned. Our study design provides insights into the time course of the acquisition of both kinds of associations, making possible observations that go beyond the existing work.

It is also important to note that we already ruled out a potential confound in the present study. The difference between ordinal-only and new-location trials also reflects a difference in the overall frequency with which the target appeared at these locations. One might argue, therefore, that any difference between ordinal-only and new-location items in these sequences reflects simple knowledge of where the target appeared more often. To rule out this alternative explanation, in the Schuck et al.’s study (2011, Experiment 2) we varied whether the ordinal-only item appeared at its correct or at an incorrect serial position within the new-location trials. Participants found targets faster when they appeared at their correct versus incorrect serial position. We did not use this method here because it involves showing fixed sequence target screen positions at the wrong serial position and in consequence might add to a potential unlearning of position-item associations (or the attentional down-weighting of these associations; cf. below the discussion of the model by Kruschke, 2003). Additionally, the results we obtained for the control condition analysis basically rule out frequency based knowledge as a main cause of the observed effects.

One main finding of the present study was the differential development of item-item versus position-item associations. Position-item associations developed quickly. They influenced ordinal-only trials already after five learning blocks, whereas item-item associations did not. However, in the long run, the relative impact of position-item associations on performance decreased while the relative impact of item-item associations seemed to remain stable. In a similar vein, practice-related changes in the impact of different forms of representation on performance have been documented before in category learning (e.g., rule- and exemplar knowledge; Johansen & Palmeri, 2002) and sequence learning entailing effector-dependent versus effector-independent sequence knowledge (e.g., Berner & Hoffmann, 2008, 2009). It is implausible that one representation can be easily deleted once a second representation becomes available during training (e.g., Shiu & Chan, 2006). Rather, it is conceivable that the acquisition of associations of one form of sequence knowledge comes close to an asymptote relatively early in training while another form of sequence knowledge only later reaches an asymptote. By this account, the relative impact on performance of one form of sequence knowledge can decrease over training without one having to assume that association strength pertaining to either form of sequence knowledge decreases. Rather, differences in the deceleration of strengthening of associations would suffice. Furthermore, there are accounts that back up learning of associative weights by attentional learning. For instance, Kruschke (2003) proposed a learning model that quickly reduces prediction errors by shifting attention away from cues that currently lead to wrong predictions while leaving associations intact. It is thus conceivable that ordinal position knowledge remains intact later in practice, but loses impact on performance because as it no longer comes to use as attention is shifted away from its cues (i.e., the fixation cross and the longer pause).

One particular important implication of our findings refers to the existence of a positional code with which associations can be formed. We believe that the nature of this serial position code is at the heart of the investigation of serial position-item associations and warrants further investigations. Despite many studies on the neural coding of rank order (for a review, see Tanji, 2001), the nature of this code remains a matter of debate. Some behavioral studies have targeted the question whether a positional code represents temporal or order information, with the results favoring the latter (Maybery, Parmentier, & Jones, 2002; Ng & Maybery, 2005), whereas other studies have stressed the existence of both (Bengtsson, Ehrsson, Forssberg, & Ullen, 2004). Of course, the representation might differ for different situations. For example, Marshuetz and colleagues (Marshuetz, Smith, Jonides, DeGutis, Chenevert, 2000) found brain areas involved in order processing in a serial working memory task that coincide with areas of number representation (Jacob & Nieder, 2008). The implication that the position code in serial working memory is a number code might only be true for tasks that involve conscious awareness, enabling “internal counting”. To disentangle such different possibilities and compare them between tasks involving explicit versus implicit memory, more groundwork is needed. By introducing the novel methodology to study the representation of serial order in implicit memory and by pointing toward some of the major issues, we hope to have provided a starting point for further investigations.

## Footnotes

1 Note that the study also included another type of transfer sequence. This condition served as a control condition and did not provide additional insights. Therefore, we excluded it from our analyses. There, sequences were constructed by using three target screen locations from the random sequence (*random-transfer trials*) and one target screen location from the fixed sequence at its correct serial position (*ordinal-random trials*). These target screen locations were chosen randomly from all possible random sequence items within one list and appeared equally often. Target screen locations in the random sequences cannot have strong item-item or position-item associations (due to the weak statistical structure). A 2 (condition: *random-transfer trials* vs. *ordinal-random trials*) by 9 (transfer block) repeated measures ANOVA was utilized to analyze the data. In line with features we described above (weak sequential structure in the random sequences should lead to weak interference effects), we found no main effect of condition, *F*(1, 20) = 2.50, *p* = .13. We also found a significant interaction between Block and Condition, *F*(8, 160) = 2.09, *p* =.04, and a main effect of block, *F*(8, 160) = 38.90, *p* < .01.
